# Understanding the life experiences of people with multiple complex needs: peer research in a health needs assessment

**DOI:** 10.1093/eurpub/ckab142

**Published:** 2021-08-26

**Authors:** Jill M Harland, Emma A Adams, Sophie Boobis, Mandy Cheetham, Alice Wiseman, Sheena E Ramsay

**Affiliations:** 1 Population Health Sciences Institute, Newcastle University, Newcastle Upon Tyne, UK; 2 Research and Evaluation, CRISIS, London, UK; 3 Department of Nursing, Midwifery and Health, Northumbria University, Newcastle upon Tyne, UK; 4 Public Health Department, Gateshead Council, Gateshead, UK

## Abstract

**Background:**

Multiple complex needs (MCN) describe a population experiencing a combination of homelessness, substance use, offending and/or mental ill-health. Using peer researchers, this study aimed to explore the perspectives of individuals with lived experience of MCN with regards to (i) issues leading to MCN and (ii) key intervention opportunities.

**Methods:**

As part of a health needs assessment in Gateshead (North East England), trained peer researchers interviewed 27 adults (aged ≥18 years) with experience of MCN, identified using purposive sampling methods. Peer researchers designed a topic guide for interviews which were audio recorded and thematically analyzed.

**Results:**

Interviewees reported adverse childhood experiences leading to MCN including abuse, bereavement, parental imprisonment, family break-up and inadequate support. Mental ill-health, substance use, poverty, early experiences of unstable housing and acute homelessness were identified as major precedents for adulthood experiences of MCN. Between 16 and 20 years, access to housing, social and mental health support was perceived as having the potential to prevent circumstances worsening. Individuals perceived removing barriers to mental health, housing and welfare and financial supports could help.

**Conclusions:**

This study highlights the perceived role austerity, adverse childhood events and current service provision have in current and future experiences of MCN. Individuals expressed a need for future interventions and support to be judgement free and provided by workers who are educated about MCN and related adversity. Involving peer researchers and individuals with experience of MCN in future research and service provision could ensure appropriate measures and supports are put in place.

## Introduction

Multiple complex needs (MCN) is a broad definition and one of a number of terms (such as severe and multiple disadvantage and multiple exclusion homelessness) seeking to identify a population experiencing co-occurring issues of homelessness, substance use, crime and mental health problems.^1–3^ This presence of overlapping vulnerabilities is associated with extreme health inequalities. In England, 58 000 people a year face all three overlapping issues (homelessness, substance use and offending); alongside nearly universally present mental ill-health and poverty.^4^ This number rises to an estimated 586 000 people when looking at those who receive services across all three domains (homeless, substance use and offending). Experiences of disadvantage do not occur uniformly across England, rather northern urban areas, some coastal areas and certain areas of London have prevalence rates two to three times the national average.^4^ With on-going rises in homeless figures,^5^ prolonged austerity,^6^ the roll out of universal credit^7^ and significantly reduced local authority budgets,^8^ the number of people affected by MCN in England is likely to rise. It is estimated that an individual with MCN, including homelessness, costs the health and social system around £19 000 per annum, which is four to five times higher than the average person.^4^ These stark figures should be considered against evidence demonstrating the return on investment from public health interventions^9^ and MCN prevention efforts/interventions^10^^,^^11^ to address wider determinants of health.

MCN is a subject of considerable interest, particularly within homelessness, substance use, and crime research and policy and practice areas. Within areas of greater deprivation and austerity, there is clustering of substance use and crime,^12^ which perpetuates experiences of MCN. Numerous studies suggest that MCN issues are progressive, rooted in childhood and linked to underlying social and structural factors, outside of the control of the individual, and many the result of social exclusion.^13–16^ These factors include histories of childhood trauma, poor family relationships, poverty, social disadvantage and poor educational experiences. Social exclusion is a multi-dimensional process where there is a degree of activity external to the individual and helps us understand some of the structural inequalities underpinning higher rates of crime and substance use.^12^ Through recognizing the role social exclusion, social harm and other structural inequities have in underpinning experiences of MCN,^12^^,^^17^ there is a need to ensure that public health responses are designed to reduce the risk and impact of MCN. Furthermore, involvement of individuals experiencing MCN is essential for developing effective interventions.^18^ However, not enough research or service development involves individuals with lived experience of MCN; thus, their insights into effective responses are missing. Relatively little health service research has used peer research methods, where people with lived experience are trained and involved in research to gain access to marginalized populations. This can provide more relevant and inclusive research informed solutions and elicit unique perspectives because of the shared experiences and ‘insider’ status peer researchers bring to discussions.^19^

This study explores this through co-conducting a health needs assessment (HNA) with peer researchers (individuals with lived experience of MCN) within the Gateshead Metropolitan Borough Council (GMBC) in the North East of England. A HNA is a systematic method of gathering and synthesizing evidence to identify unmet health and healthcare needs of a population—in this case, individuals experiencing homelessness and MCN. Local authorities have a recognized role in influencing the wider determinants of health and a statutory duty to address health inequalities.^20^ Through using peer research methods, this health services research aims to explore the perspectives of individuals with lived experience of MCN with regards to (i) issues leading to MCN and (ii) key intervention opportunities.

## Methods

The study was co-produced with GMBC, Fulfilling Lives Newcastle Gateshead (FLNG) (a voluntary organization working to improve outcomes for those facing MCN) and academics from Fuse (Centre for Translational Research in Public Health).

Individuals with current experience of homelessness and MCN were recruited by FLNG through an existing service user group to undertake an accredited research skills training course developed by FLNG. The ‘peer research’ training was designed to equip individuals with the skills to take ownership of a research project including its design and delivery. Two trained peer researchers participated in all aspects of the study, including study design, development of the interview guide, participant recruitment, data analysis and writing up findings for the HNA. The peer researchers conducted individual semi-structured face-to-face interviews (*n* = 27) to enable adults (aged ≥18 years) with lived experience of MCN to share their experiences.

Interview participants were recruited using a purposive and targeted sampling approach to identify adults aged ≥18 years in Gateshead experiencing homelessness and MCN who were not necessarily engaging with services. The 27 people recruited met this inclusion criteria and were identified via links with community organizations and the peer researchers’ own social networks. No individuals who expressed interest were excluded or left the study. Research and development at the GMBC reviewed and provided ethical approval as part of the HNA. Potential participants were given an information sheet, assured of anonymity and confidentiality and reminded they had the option to withdraw at any time. Participants had the opportunity to ask questions before taking part and provided written informed consent. Participants’ demographic information was exclusive to gender and age to maintain anonymity; specifics on substance use and other social determinants of health were not recorded. An interview guide was devised and piloted by the peer researchers. Interviews focused on three themes: (i) issues leading to MCN outcomes; (ii) when support could have made a difference; and (iii) what type of support could have made a difference. Interviews took approximately 45 min and were digitally recorded and transcribed. Any personal information was removed and transcriptions were checked for accuracy. No incentives were offered to the participants. Responses for each interview were recorded on an excel spread sheet. Inductive thematic analysis was undertaken by the peer researchers and SB^21^ using the pre-existing interview themes. This involved identifying emerging codes and categorizing them across the data. Coded extracts were then collated into themes and sub themes and discussed iteratively to determine whether the individual themes accurately reflected the meanings of participants. Researchers refined themes, agreed on how each theme addressed the research questions and identified quotes.

## Results

All 27 adults (14 women and 13 men) participated and were interviewed by two peer researchers. Mean age of the participants was 32 years, ranging from 22 to 55 years.

Findings below are presented according to the three main themes.

### Issues leading to and accentuating MCN outcomes

Participants perceived a range of similar experiences which interacted and accumulated in a detrimental and dynamic way over time to exacerbate negative circumstances. These were felt as leading to recurrent and circular experiences of homelessness and MCN.

#### Experiences of adversity

Participants shared experiences of adversity and austerity across their lifespan. Most participants identified childhood as the time when issues leading to homelessness and MCN began. Adverse experiences included childhood abuse, neglect, parental domestic abuse, bereavement, parental mental health issues, parental imprisonment, being a young carer, family break up, homophobia and being in care.

*‘mam and dad in prison, addiction and mental health. As a kid I ran away from foster homes and care as was not treated properly* *’*

When discussing adversity in the context of adulthood, participants perceived financial exclusion and poverty as reasons why individuals experienced MCN. People’s financial difficulties were caused by challenges with managing on limited budgets, which were precipitated by a spiralling cycle of deteriorating mental health, substance use, homelessness and crime.

*‘made redundant due to mental health and not being able to keep up rent on flat* *’*

*‘I didn’t know how to handle money, got evicted, no support* *’*

Adverse childhood experiences alongside later life experiences of poverty led to individuals becoming stuck in a cycle of MCN where they were unable to address underlying issues.

#### Mental health, substance use and access to services

Poor mental health was perceived as a common antecedent for MCN, often linked to adverse childhood experiences.

*‘at 15/16 social workers and workers at refuge didn’t help with my mental health* *’*

*‘there needs to be some support for children being brought up by unstable parents in domestic violent homes with mental health* *’*

Substance use (sometimes co-occurring with mental ill-health called dual diagnosis) was also perceived as negatively impacting experiences of MCN. Participants described being refused help because of their use of drugs, feeling judged and stigmatized. Such experiences reduced trust in services and led to a lack of motivation to seek support.

*‘I felt like they didn’t care. They said I was complicated and couldn’t help as I had mental health and addiction. So why would I trust them again* *’*

*‘I needed to be made more aware of addiction and recovery and not judged and discriminated against* *’*

Participants identified difficulty accessing support from mental health services, social care, housing, general practice, secondary care and dental care. Barriers included: not being able to register or maintain appointments due to homelessness, not meeting referral criteria for mental health and housing because of substance use and an inability to complete referral forms because of difficulties reading and writing. These barriers led to worsening of experiences and isolation from support.

*I can’t attend appointments due to mental health and am blamed for that and isolated more and helped less*

#### Homelessness and vulnerable housing

Experiences of homelessness occurred early on in the MCN journey, including staying in hostels, sofa surfing, living on streets and insecure housing. First time homelessness experiences were mostly reported to take place between 16 and 30 years of age. Average time spent homeless or vulnerably housed was 3 years and varied from 6 months to 20 years. There were many accounts of being placed in hostels that were intimidating environments where individuals were exposed to bullying, substance use and criminality. Individuals shared experiences of selecting their housing options based on which felt safer.

*I had nowhere safe to live so sofa surfed and was street homeless as this was safer than foster care*

*was terrified by other tenants bullying and using substances felt safer sofa surfing and engaging in one-night stands for somewhere to stay and sleeping on the streets*

Over half of participants described being discharged from hospital homeless; one-fifth had left prison without accommodation and there were accounts of leaving care without appropriate housing. Participants highlighted the lack of accommodation when leaving critical contacts with the health, social and criminal justice system increased chances of experiencing MCN.

### Timing of support to make a difference

When reflecting on opportunities to intervene, participants highlighted that future and current interventions should be available around late teens and early 20s.

#### Early years

Late teens and early 20s were identified as critical periods when help to address physical, social and mental health needs may have prevented MCN experiences.

*helping me when I was younger could have saved me a lot of suffering and further physical and mental health problems and trauma*

*Education and help at 14 when discharged from hospital could have given me choices and opportunities which could have alleviated my homelessness*

Not being listened to at an early age and not receiving support from services was a repeated theme.

*At 13 no one asked what was going on and why I was behaving as I was, stepdad abusing me and then sent me into care. I was branded a problem kid but needed help not punishment*

For those leaving the care system, transition to adulthood meant changes in care and support arrangements at a time when help was critical.

### Changing support to improve care for individuals experiencing MCN

Participants recognized that addressing the time when interventions were offered would be insufficient for addressing MCN without changing the actual support available to improve experiences.

#### Support focused on mental health, finance and welfare, and housing

Participants highlighted that there was a gap in current support when it came to mental health, housing and finance. Mental health support was the most commonly cited intervention that would have prevented homelessness and MCN issues. Participants said emotional and psychological support was needed to help deal with challenging family and social circumstances.

*‘* *if as a young child I had been listened to and received appropriate mental health care rather than being sent away to inappropriate psychiatric institutions I believe this could have helped me deal with issues and prevent my mental health deteriorating and me from spiralling into addiction and self-destructive behaviours* *’*

Many participants said financial and welfare advice could have prevented their situation deteriorating. They identified the need for advice on debt, paying rent, completing forms, managing money, paying bills, welfare support and employment and education opportunities.

‘*help to manage money and to pay the bills’*

Without substantial finance and mental health support, many individuals found themselves needing housing support. The need to have a ‘home’, permanence and housing choice was articulated. Specifically, an environment where they felt safe, secure and were not exposed to issues they were trying to overcome (such as substance use or criminal behaviour). Participants said age-appropriate supported housing was needed, with protection from bullying behaviour.

*‘* *appropriate safe and long-term housing would have made it better* *’*

*‘we need good hostels, with no drugs, offending or crime, help to get a job and to keep families together* *’*

There was a perception that having a long-term home was essential to allowing individuals to break their cycle of MCN.

#### Educating workers and providing judgement-free support to enhance experiences

Discussions around how current services could be improved highlighted a need to enhance training so that individuals are aware of issues leading to and faced by individuals experiencing MCN, while also emphasizing that any support offered needs to be free from judgement and stigma. It was suggested that teachers, doctors and mental health workers needed to be more aware of signs of childhood difficulty and be able to intervene.

*‘I think teachers, mental health professionals and housing workers need training in multiple and complex needs, dual diagnosis and warning signs of abuse and addiction, people need to not be afraid of asking young people why they are struggling and not shame them for being mentally ill* *’*

Through increasing awareness, participants perceived that interventions could take place earlier on.

There was a perceived gap between what was being offered and what was needed and sometimes this created unnecessary barriers. Participants suggested staff needed training around MCN issues and needed to involve people with lived experience in designing care packages. Services needed to make it easier for people to register and attend.

*‘* *educate staff from front-line upwards, use people with lived experience, stop barriers which prevent those who are homeless from accessing health care such as postcode requirements* *’*.

Irrespective of the kind of support, the support needed to be respectful and non-judgemental. Participants consistently shared experiences of not being listened or given a chance as they were judged the moment they walked through the door.

*‘give people a chance and listen, I am not a bad person but people think I am straight away because of my past and criminal record* *’*

These previous experiences deterred people from seeking help as they assumed they would face the same judgement and stigma.

*‘they made me feel like I was wasting their time and they did not want to help or have time so I didn* *’t ask anymore* *’*

There was a perception that by training staff about the complex issues faced by individuals experiencing MCN, experiences accessing support would be improved.

## Discussion

By adopting a peer research approach in completing a routine public health evaluation (a HNA), we have been able to obtain insights into the perceived problems and solutions for tackling MCN from perspective of individuals experiencing MCN. These action-oriented insights stemmed from questions and issues that are important to individuals experiencing MCN. The peer research approach adopted in this study enabled engagement with marginalized individuals whose voices may not typically appear at the forefront in a HNA. Insights shared by individuals experiencing MCN into the perceived issues leading to MCN and opportunities for intervention suggest a need to focus on negative experiences in childhood and broader austerity. Furthermore, the perspectives shared suggest current service provision requires changes in its approach and offer.

### Childhood factors

Many of the issues perceived by individuals with MCN as leading to or accentuating experiences of MCN are commonly reported in literature. Studies from the UK and other countries have shown that adverse experiences in early life have a lasting effect on physical and mental health in adulthood.^22–24^ Participants shared a myriad of adverse childhood experiences when discussing perceived causes of adulthood MCN; however, the emphasis on childhood as a critical time when effective support could have prevented difficulties escalating cannot be ignored. Recognizing signs of childhood difficulty and providing timely, sensitive support was critical for preventing MCN. Poor mental health often linked to adverse childhood experiences was commonly described as precipitating MCN. Participants identified critical time periods that led to MCN outcomes across the life course: early teens, transition to adulthood and institutional discharge from prison and hospital. Our findings emphasized the need to recognize and respond to mental health problems in childhood.

### Austerity

The longer a person experiences homelessness, particularly from young adulthood, the more risks there are to health and wellbeing, increasing chances and experiences of MCN.^25^ A review of policies and research on social exclusion identified the most intractable problems faced by those with MCN tend to be poverty and worklessness.^26^ Risks to vulnerable people with complex lives have heightened following austerity and welfare reform including the roll out of universal credit, prolonged austerity and cuts to public services.^7^^,^^27^^,^^28^ The need for upstream policy measures to reduce material poverty and prevent extreme health and social inequalities from occurring is clear.^18^ A review about marginalized and excluded populations^18^ found that multicomponent interventions involving individual care coordination or case management had higher effectiveness than stand-alone interventions.

### Service provision

Homelessness, poverty, substance use and having a criminal record were barriers to receiving support. Coupled with feeling judged and stigmatized, negative experiences of access perpetuated a lack of trust in services and deeper exclusion. Participants highlighted the need for services to have more flexible access arrangements and non-judgemental approaches which ‘*see the person not the problem*’. A key finding was the damaging role that poor housing and/or inadequate service responses play in exacerbating problems and reducing help seeking behaviour. In terms of types of support needed to reduce MCN, our study found that appropriate, timely access to mental health support, safe and secure housing, and financial support were important. Our study corroborates research showing that services fail to adequately address MCN and individuals often fall through the gap between community, voluntary and statutory services.^29^ The need for support that is sensitive to an individual’s own perception of need and for services to improve their understanding, sensitivity and response to MCN through prioritizing training to equip staff was articulated.

### Strengths and limitations

The strength of this study lies within the use of peer research approaches. Through listening to individuals with experience of MCN, the HNA was able to explore issues that directly affect their community. This is particularly novel within the context of HNAs, which are meant to identify unmet needs and inform service development. Creating an approachable data collection environment, providing an insider status and contributing unique insights were some of the positive benefits peer researchers contributed to the study. The insights specific to opportunities for service provision take into consideration the lived experience, which can lead to more equitable service delivery and engagement.

Peer research bias could have arisen from finding service users close enough to the subject area to bring real experience whilst also capable of sufficient detachment to enable reflective and objective analysis.^30^ However, the peer researchers in our study underwent training to explore such issues and were supported through regular reflective practice and discussions within the team in line with good research practice. Our study was conducted in the North East of England and it is possible that the conclusions drawn relate to the experiences, social context of the participants and services in that area. We recognize that the experiences of participants in our study may not represent the experiences of all people experiencing MCN.

## Conclusions and implications

Our study highlights the role adverse childhood events and austerity have in future experiences of MCN from the perspective of those with lived experience. It re-focuses the conversation on intervention opportunities towards highlighting absent, but much needed support that is judgement-free and aware of the complex issues faced by those experiencing MCN. Through incorporating peer researchers in routine practices, the study demonstrates the opportunity local health authorities and broader health services have to ensure aims, objectives and outcomes align with the needs of the population. Further research is needed to develop effective whole system approaches to preventing MCN across health, housing, social and education sectors.

## Funding

No funding was received for this work. E.A.A., M.C. and S.E.R. are members of Fuse, the Centre for Translational Research in Public Health (www.fuse.ac.uk). Fuse is a UK Clinical Research Collaboration (UKCRC) Public Health Research Centre of Excellence. Funding for Fuse from the British Heart Foundation, Cancer Research UK, National Institute of Health Research, Economic and Social Research Council, Medical Research Council, Health and Social Care Research and Development Office, Northern Ireland, National Institute for Social Care and Health Research (Welsh Assembly Government) and the Wellcome Trust, under the auspices of the UKCRC, is gratefully acknowledged. E.A.A. is supported by the National Institute for Health Research (NIHR) School for Public Health Research (SPHR) Pre-doctoral Fellowship, Grant Reference Number PD-SPH-2015. This research is supported by the National Institute of Health Research (NIHR) Applied Research Collaboration (ARC) for the North East and North Cumbria (NENC). The National Institute for Health Research (NIHR) is the nation’s largest funder of health and care research and provides the people, facilities and technology that enables research to thrive. Find out more about the NIHR. The views expressed are those of the author(s) and not necessarily those of the NIHR or the Department of Health and Social Care.

*Conflicts of interest: None declared.*

**Key points**

- Engaging peer researchers allows for action-oriented findings to address the issues that are most important to individuals experiencing MCN—this is particularly relevant for health needs assessments, which identify unmet need and inform service provision.
- Effective support in late teen and early adulthood could be one of the best ways to prevent MCN upstream.
- Prioritizing training to equip staff in all services to recognize people at risk of MCN and being sensitive to their issues could improve current service provision.

## References

[1] Aldridge RW , Story A, Hwang SW, et al Morbidity and mortality in homeless individuals, prisoners, sex workers, and individuals with substance use disorders in high-income countries: a systematic review and meta-analysis. Lancet 2018;391:241–50. 29137869 10.1016/S0140-6736(17)31869-X PMC5803132

[2] Dobson R. Complex needs in homelessness practice: a review of ‘new markets of vulnerability’. Housing Studies 2019;1–27.

[3] Rosengard A , Laing I, Ridley J, Hunter S. A Literature Review on Multiple and Complex Needs. Edinburgh, Scotland: Scottish Executive Social Research, 2007. Available at: https://www.webarchive.org.uk/wayback/archive/20170112055441/, http://www.gov.scot/Publications/2007/01/18133419/15 (August 2020, date last accessed).

[4] Bramley G , Fitzpatrick S, Edwards J, et al Hard Edges: Mapping Severe and Multiple Disadvantage (England). London, UK: Lankelly Chase Foundation, 2015. Available at: https://lankellychase.org.uk/wp-content/uploads/2015/07/Hard-Edges-Mapping-SMD-2015.pdf (August 2020, date last accessed).

[5] Fitzpatrick S , Pawson H, Bramley G, et al The Homelessness Monitor: England 2019. London, UK: Crisis, 2019. Available at: https://www.crisis.org.uk/media/240419/the_homelessness_monitor_england_2019.pdf (August 2020, date last accessed).

[6] British Medical Association. Cutting Away at Our Children’s Futures: How Austerity is Affecting the Health of Children, Young People, and Families. London, UK: BMA, 2016. Available at: https://www.bma.org.uk/media/2060/cutting-away-at-our-childrens-futures-austerity-child-health-guuk-2016.pdf (September 2020, date last accessed).

[7] Cheetham M , Moffatt S, Addison M, Wiseman A. Impact of universal credit in North East England: a qualitative study of claimants and support staff. BMJ Open 2019;9:e029611. 10.1136/bmjopen-2019-029611 PMC6615785 31272984

[8] British Medical Association. Feeling the Squeeze: The Local Impact of Cuts to Public Health Budgets in England.London, UK: BMA, 2018. Available at: https://www.bma.org.uk/collective-voice/policy-and-research/public-and-population-health/public-health-budgets (August 2020, date last accessed).

[9] Masters R , Anwar E, Collins B, et al Return on investment of public health interventions: a systematic review. J Epidemiol Community Health 2017;71:827–34. 28356325 10.1136/jech-2016-208141 PMC5537512

[10] Public Health England. *Evidence Review: Adults with Complex Needs (With a Particular Focus On Street Begging and Street Sleeping).* London, UK: PHE publications, 2018. Available at: https://assets.publishing.service.gov.uk/government/uploads/system/uploads/attachment_data/file/680010/evidence_review_adults_with_complex_needs.pdf (September 2020, date last accessed).

[11] The Calouste Gulbenkian Foundation, Making Every Adult Matter. Individuals with multiple needs: the case for a national focus. 2015. Available at: http://meam.org.uk/wp-content/uploads/2015/05/Individuals-with-multiple-needs-the-case-for-a-national-focus.pdf (August 2020, date last accessed).

[12] Seddon T. Drugs, crime and social exclusion: social context and social theory in British drugs–crime research. Br J Crimnol 2006;46:680–703.

[13] Mabhala MA , Yohannes A, Griffith M. Social conditions of becoming homelessness: qualitative analysis of life stories of homeless peoples. Int J Equity Health 2017;16:150. 28830515 10.1186/s12939-017-0646-3 PMC5568348

[14] Bramley G , Fitzpatrick S. Homelessness in the UK: who is most at risk? Housing Studies 2018;33:96–116.

[15] McDonagh T. Tackling Homelessness and Exclusion: Understanding Complex Lives. York, UK: Joseph Rowntree Foundation, 2011. Available at: https://www.jrf.org.uk/sites/default/files/jrf/migrated/files/homelessness-exclusion-servicessummary.pdf (August 2020, date last accessed).

[16] Public Health Wales. Adverse childhood experiences and their impact on health-harming behaviours in the Welsh adult population. Public Health Wales NHS Trust, 2016. Available at: http://www2.nphs.wales.nhs.uk:8080/PRIDDocs.nsf/7c21215d6d0c613e80256f490030c05a/d488a3852491bc1d80257f370038919e/$FILE/ACE%20Report%20FINAL%20(E).pdf (August 2020, date last accessed).

[17] Hillyard P , Pantazis C, Tombs S. Beyond Criminology: Taking Harm Seriously. Winnipeg, Canada: Pluto Press, 2004.

[18] Luchenski S , Maguire N, Aldridge RW, et al What works in inclusion health: overview of effective interventions for marginalised and excluded populations. Lancet 2018;391:266–80. 29137868 10.1016/S0140-6736(17)31959-1

[19] Terry L , Cardwell V. Refreshing Perspectives: Exploring the Application of Peer Research with Populations Facing Severe and Multiple Disadvantage. London, UK: Revolving Doors Agency & Lankelly Chase, 2016. Available at: http://www.revolvingdoors. org.uk/file/1849/download?token=Yi0tjhmo (August 2020, date last accessed).

[20] Local Government Association. A matter of justice: local government’s role in tackling health inequalities. 2016. Available at: https://www.local.gov.uk/sites/default/files/documents/1.42%20public%20health%20inequalities_web.pdf (August 2020, date last accessed).

[21] Braun V , Clarke V. Using thematic analysis in psychology. Qual Res Psychol 2006;3:77–101.

[22] Marmot M. *Fair Society, Healthy Lives: The Marmot Review: Strategic Review of Health Inequalities in England Post-2010*. 2010. London: Institute of Health Equity, ISBN 9780956487001.

[23] House of Commons Science and Technology Committee. Evidence-based early years intervention. London House of Commons, 2018. Available at: https://publications.parliament.uk/pa/cm201719/cmselect/cmsctech/506/506.pdf (August 2020, date last accessed).

[24] Hughes K , Bellis MA, Sethi D, et al Adverse childhood experiences, childhood relationships and associated substance use and mental health in young Europeans. Eur J Public Health 2019;29:741–7. 30897194 10.1093/eurpub/ckz037 PMC6660110

[25] Public Health England. Homelessness: applying all our health June 6, 2019. Available at: https://www.gov.uk/government/publications/homelessness-applying-all-our-health/homelessness-applying-all-our-health#introduction (August 2020, date last accessed).

[26] Joseph Rowntree Foundation. Reducing Poverty in the UK: A Collection of Evidence Reviews. London, UK: Joseph Rowntree Foundation, 2014. Available at: https://www.jrf.org.uk/report/reducing-poverty-uk-collection-evidence-reviews (August 2020, date last accessed).

[27] Human Rights Council. Report of the Special Rapporteur on extreme poverty and human rights on his visit to the United Kingdom of Great Britain and Northern Ireland. United Nations, 2019. Available at: https://documents-dds-ny.un.org/doc/UNDOC/GEN/G19/112/13/PDF/G1911213.pdf?OpenElement (August 2020, date last accessed).

[28] Hood A , Waters T. Living Standards, Poverty, and Inequality in the UK: 2017–18 to 2021–22. London, UK: Institute for Fiscal Studies, 2017. Available at: https://www.ifs.org.uk/uploads/publications/comms/R136.pdf (August 2020, date last accessed).

[29] Anderson S. Complex Responses: Understanding Poor Frontline Responses to Adults With Multiple Needs: A Review of the Literature and Analysis of Contributing Factors. London, UK: Revolving Door Agency, 2011. Available at: http://www.revolving-doors.org.uk/file/1796/download?token=pZa0cCm3 (August 2020, date last accessed).

[30] Burns S , Schubotz D. Demonstrating the merits of the peer research process: a northern Ireland case study. Field Methods 2009;21:309–26.

